# Examining ecosystem (dis-)services on liver fluke infection in rural Northeast Thailand

**DOI:** 10.1186/s40249-023-01079-y

**Published:** 2023-04-19

**Authors:** Yi-Chen Wang, Andrea Law, Jutamas Namsanor, Paiboon Sithithaworn

**Affiliations:** 1 Department of Geography, National University of Singapore, 1 Arts link, Block AS2, 117568 Singapore, Singapore; 2 Department of Parasitology and Cholangiocarcinoma Research Institute, Faculty of Medicine, Khon Kaen University, 123 Mittraphap Rd, Mueang Khon Kaen District, 40002 Khon Kaen, Thailand

**Keywords:** Liver fluke infection, One health, Neglected tropical disease, Cultural ecosystem service, Ecosystem disservice, Human-environment interaction

## Abstract

**Background:**

The direct reliance of humans on and their interactions with freshwater ecosystems in the Lower Mekong Basin have given rise to parasitic infections, which is particularly prevalent in Northeast Thailand where raw fish consumption is practiced. This study examined the interactions between environments, ecosystem (dis-)services, human raw fish consumption habits, and raw fish dish sharing on liver fluke infection risk.

**Method:**

Water fecal contents and the first intermediate snail host were sampled between June and September of 2019. One hundred twenty questionnaires were surveyed in two villages of different environmental surroundings, one next to a river and the other located inland, in Northeast Thailand. Multivariate regression analyses using linear mixed effect models assessed the influence of social, behavioral and perceptual factors on raw fish consumption frequency, willingness to avoid consumption and liver fluke infection status. Social network analysis compared the degree of raw fish dish sharing between the villages and assessed the probable influence of connections to fish procurement locations and sharing activities on liver fluke infection risk.

**Results:**

High abundance of the first intermediate snail host and presence of fecal contamination in water could endanger both villages to ecosystem disservices of parasitic transmission. The river-side village relied more on provisioning ecosystem services than the inland village (29.7% vs. 16.1% of villages) to consume raw fish as their main source of protein. Males in both villages (64.5 and 40.4 days/year for the respective villages) are also likely to consume *koi pla* and *pla som*, higher risk fish dishes, more frequently than females (4.1 and 4.3 days/year for the respective villages). The consumption habits of both villages were driven mostly by deriving cultural ecosystem services. Participation in raw fish dish sharing activities significantly reduced the odds of an individual being willing to avoid the consumption (Odds ratio = 0.19). Network analysis suggested that river-side villagers had a more direct raw fish dish sharing interaction and they procured fish from multiple locations; these characteristics might potentially account for more liver fluke infected households in the village.

**Conclusion:**

Villagers’ raw fish consumption is driven by deriving cultural ecosystem services, and the geographic settings of the villages potentially affect villagers’ fish procurement locations and infection risk. The findings underscore the linkages between villagers and their surrounding ecosystem environments as pertinent determinants for foodborne parasitic disease risk.

**Graphical Abstract:**

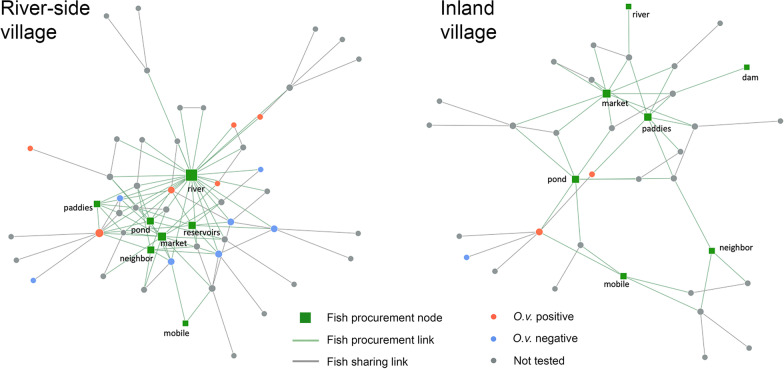

**Supplementary Information:**

The online version contains supplementary material available at 10.1186/s40249-023-01079-y.

## Background

Healthy ecosystems are vital for the survival of humans and animals. Nearly 75% of emerging human infectious diseases in the past three decades originate from animals [1, 2]. This calls for a One Health approach, which recognizes the close linkage and interdependence among the health of humans, animals, and the wider ecosystem environments [3]. There has been growing interests in promoting integrative, transdisciplinary research on ecosystem-health relationships due to the complexities in both positive and negative health impacts and the interactions with socio-economic and ecological factors [4, 5]. Consequently, new ways of tackling infectious diseases are needed, to integrate concepts and methods from different fields, particularly social and ecological sciences, in addition to biomedicine and public health [6].

The linkages between ecosystems and human well-being can be depicted through an integrated framework of ecosystem services and ecosystem disservices [7]. Defined as the benefits people obtain from ecosystems, ecosystem services include provisioning services such as food and water, regulating services such as climate regulation, cultural services providing recreational and spiritual benefits, and supporting services such as nutrient cycling [8]. The reliance on these benefits also potentially endangers humans to adverse ecosystem functions. The ecosystem disservices concept calls attention to the negative effects of ecosystems on human well-being [9]. Disservices can result from the functioning of undisturbed ecosystems or anthropogenic activities, such as forest fires, wild animal attacks and zoonotic diseases transmitted to humans [7, 9]. Particularly, the disservice of human exposure to infectious diseases via pathogens has been underscored as of global concern [10].

The co-existence of ecosystem services and disservices manifests in freshwater ecosystems. Humans depend much on freshwater provisioning services (e.g., domestic and farm water usage and fisheries). Population growth and economic development have quickened anthropogenic ecosystem changes through dam constructions and stream diversion to stabilize and deliver water supplies [11]. Such large-scale water resources development projects, albeit contributing to greater water and food security, have impaired health with water-related diseases. Extensive literature has investigated the outbreaks or increased endemicity of freshwater system related infectious diseases over the past half century [12–15]. Northeast Thailand (or *Isan* in Thai), a region economically dominated by agriculture, is no exception. Freshwater ecosystems are central to livelihood activities of the communities in the region, yet also provide habitats for disease hosts to thrive. Of particular public health concern in *Isan* and broadly, the Lower Mekong River Basin in Southeast Asia, is the foodborne parasitic infection of liver fluke *Opisthorchis viverrini* (*O. viverrini*). The life cycle of *O. viverrini* is completed through the freshwater ecosystems, involving *Bithynia* species snails as the first intermediate host, cyprinid freshwater fish as the second intermediate host and human as the definitive host. *O. viverrini* eggs are mainly shed in the feces of human hosts into freshwater ecosystems due to poor sanitation practice or underdeveloped sewage infrastructure. The eggs have to be ingested by *Bithynia* snails to continue the life cycle. Following multiplication in snails, hundreds of cercariae, the larval form of the parasite, can be shed by infected snails per day. When cercariae encounter cyprinid fish, they penetrate the fish scale, encyst and form metacercariae. Human become infected through the consumption of raw or undercooked fish contaminated with the metacercariae.

*O. viverrini* infection has been prevalent and persistent in the region for decades, because of the intertwined relationships between human and ecosystems. Fish, snails, and other aquatic animals from the freshwater ecosystems provide cheap animal protein for villagers [16], offering the provisioning services of food. Additionally, excess fish are often fermented for household consumption and for food sharing with kith and kin, which is a long-standing reciprocity of the rural farming culture [17, 18]. Consumption of raw fish has also been linked to the constructions of masculinity in *Isan* [19]. The deeply embedded raw fish consumption practice in *Isan* culture and the sharing of these raw and fermented dishes in social gathering and religious rituals symbolize the cultural ecosystem services. Continual derivation of these provisioning and cultural ecosystem services inevitably exposes the *Isan* communities to ecosystem disservices of high *O. viverrini* infection risk. Humans are more than just the definitive host of the parasite life cycle. At the landscape level, anthropogenic activities, especially water resources development projects, have exacerbated the impacts of ecosystem disservices. Constructions of dams and irrigation systems for agriculture and aquaculture not only expand the breeding grounds for *O. viverrini* intermediate hosts, but also provide water-connected pathways between different host habitats, leading to greater contact between hosts for disease transmission [20]. At the social interaction level, food sharing practice has been associated with the spread of foodborne pathogens across households especially in rural villages [21] (Trostle et al., 2008). Sharing raw and undercooked fish dishes potentially contaminated with *O. viverrini* thus increase the risk of *O. viverrini* infection to individuals who do not make the fish dishes. As the *Isan* and other Lower Mekong Basin rural communities indispensably rely on freshwater ecosystem services, it is pertinent to take the livelihood connection with the ecosystem into consideration, along with the human social interactions, when studying the risk of *O. viverrini* infection.

This study thus aims to examine the interactions between ecosystem environments, human raw fish consumption, and extent of raw fish dish sharing on *O. viverrini* infection risk. Using field and questionnaire surveys from two *Isan* villages of different environmental surroundings, this study addresses the following research questions. First, how do the freshwater ecosystems of village surroundings contribute to ecosystem disservices of *O. viverrini* intermediate snail host habitats and their infections? Second, what are the frequencies and reasons for human raw fish consumption, are there gender differences, and to what extent do cultural and provisioning ecosystem services affect consumption frequencies and willingness to avoid consumption? Third, do villagers have varying social interactions of raw fish dish sharing and different locations for obtaining fish, and how might these differences affect their *O. viverrini* infection risk? The incorporation of concepts of ecosystem services and disservices and analysis of social networks of food sharing and fish procurement locations will offer new insights into foodborne parasitic infection risk. These social and ecological perspectives will underscore the linkages between villagers and their surrounding ecosystem environments as pertinent determinants for disease transmission, thereby contributing to a more holistic understanding of *O. viverrini* infection risk.

## Methods

### Study setting

**Fig. 1:**
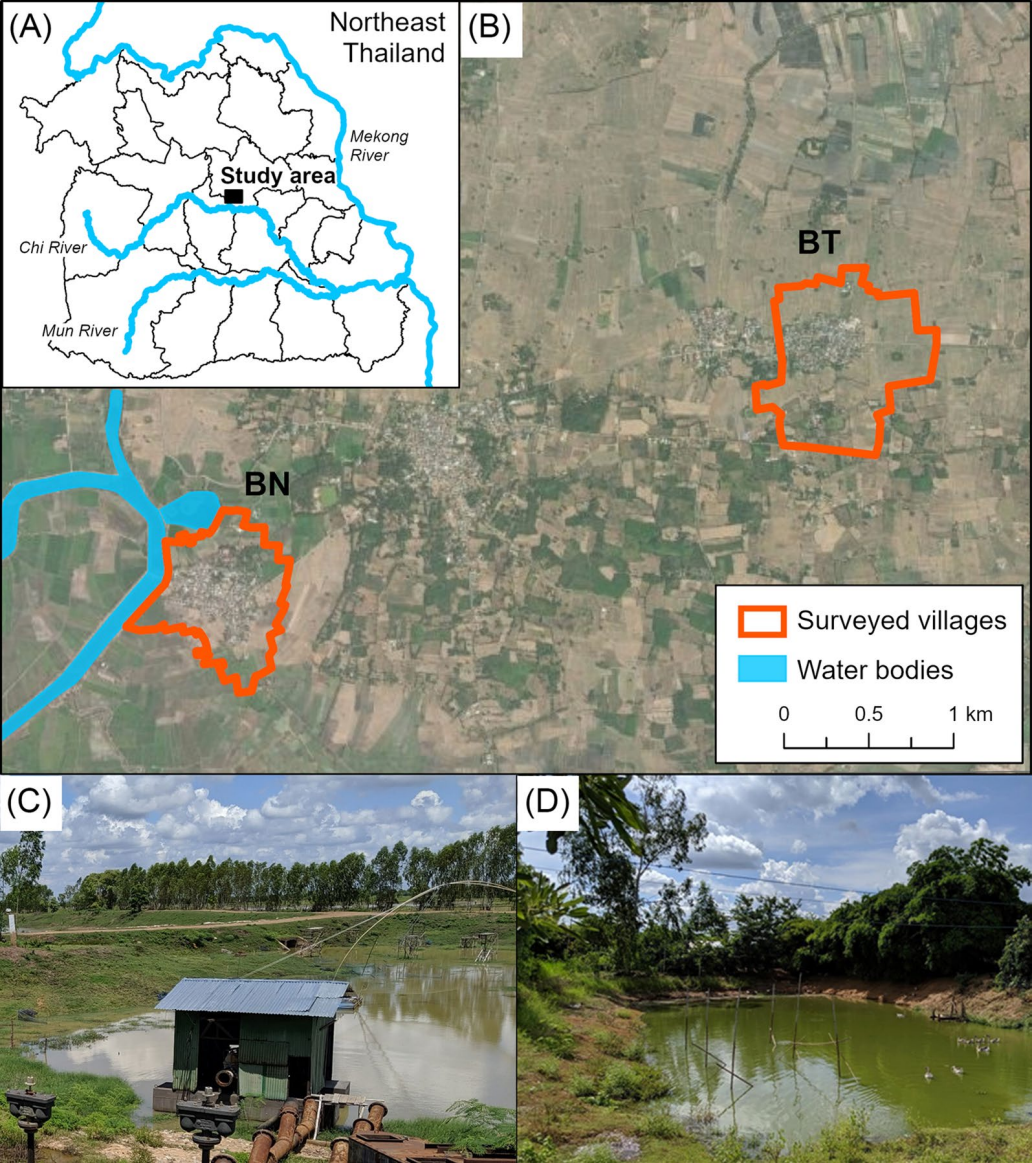
**A** Location of the study area in Northeast Thailand. **B** Surveyed villages *Ban* Nam (BN) and *Ban* Tong (BT), and their landscape surroundings. Fishing activities using **C** the ‘lift net fishing’ structure and **D** poles for ‘brushwood fishing’ are evident in the study area, showing the strong connection with the environment for ecosystem services

Northeast Thailand has been a high endemic area for human *O. viverrini* infection and also reported the highest incidence of *O. viverrini*-associated bile-duct cancer (i.e., cholangiocarcinoma) in the world [22]. The study area is located in the southern Kalasin Province, a lowland floodplain of the Chi River (Fig. 1). Prevalence of *O. viverrini* infection remained high at 37.3% in 2016 for the sub-district [23] where the two surveyed villages are situated. The study focuses on a wetland environment, with a mosaic landscape of river, pond, rice paddy, and human settlement. The products and services (e.g., water supply, rice farming and fishing) from the wetland ecosystem are central to local livelihood practices. Two villages (‘*Ban*’ in Thai), *Ban* Nam (BN) and *Ban* Tong (BT) (pseudonyms) from the area were selected, because of their differences in physical environment, and proximities to a river system. BN is adjacent to a river and a large pond, while BT is more than 4 km away from the river, surrounded by rice paddies and farmland (Fig. 1).

### Field sampling and laboratory analysis of snail and water

To investigate research question one on whether freshwater ecosystems of village surroundings contribute to ecosystem disservices of *O. viverrini* transmission, two types of samples, snail and water, were collected. Field work was done in 2019 between June and September. Sampling plots were set to include all water bodies within 100 m from the edge of the household area of each village, which comprised mostly rice paddies.

Snail sampling was performed to understand the extent to which the first intermediate *Bithynia* snail host dominated the freshwater ecosystems in the village surroundings and the parasitic infections in snails. The time search method [24] with 10-minute search was carried out by two researchers concurrently to collect as many snails as possible at each sampling plot. The researchers moved around the periphery of a water body sampling plot to collect snails by either hand-picking or using a scoop net, depending on the plot conditions. The collected snails were brought back to the laboratory for species identification to further compute their relative abundances. The standard cercarial shedding method for detecting parasitic infection in snails [25] was done for *Bithynia* snails to determine their parasitic infections. Under a light microscope, the emerged cercariae of different parasites, including *O. viverrini*, were identified by morphology according to the keys in [26]. Infection rates of different parasites in *Bithynia* snails were then calculated.

Because prior literature has shown low *O. viverrini* prevalence in the first intermediate snail host [27], water sampling was done to examine fecal contamination in the village surroundings as a proxy for potential *O. viverrini* transmission [28, 29]. Fecal contamination was measured using *Escherichia coli* concentration, as the presence of *E. coli* in water indicates potential contamination by human and other mammalian feces, which could carry parasite eggs for disease transmission. To detect *E. coli* concentration, the Colisan Easygel testing kit (Micrology Laboratories) was used for its usefulness in providing quantitative estimations on *E. coli* content in the water environment [30]. From each sampling plot, 5 ml of water was collected and poured into the testing kit, stored in an ice box, and brought back to the laboratory. Next, the samples were poured into pretreated petri dishes for incubation at 35 ℃ for 24 h. Thereafter, purple colonies resulted from the interactions between the Easygel medium and the enzymes produced by *E. coli* were counted and quantified as the colony forming units (CFUs) per 100 ml of water based on Eq. (1):

1 $$CFU\,per\,100\,ml = \frac{Number\,of\,CFU\,_{identified}}{Volume\,of\,water\,sample\, (i.e.,\,5\,ml)} \times 100.$$

### Questionnaire survey

To investigate research question two on the effect of ecosystem services on raw fish consumption habits and research question three on the influence of raw fish dish sharing on *O. viverrini* infection risk, questionnaire survey was conducted to gather information on 120 villagers’ raw fish consumption habits, sharing practices and *O. viverrini* awareness. The survey was conducted in Thai with the aid of local translators who had been introduced about the life cycle of the parasite, public health information of the infection, and the contexts of the survey questions asked. Convenience sampling was used to recruit participants based on their availability at the time of the visit and their willingness to take part in the survey. The participants were also invited to raise queries at any time during the survey should any clarification be needed regarding the questions asked.

To ensure quality control, the survey took the form of structured interviews with the questionnaire consisting of multiple sections of mostly multiple-choice questions. This ensures that the questions asked and the options provided for response were consistent across the villages. The option ‘Others’ is also provided for participants to elaborate should their answers not be found in the list of options provided. The first section of the questionnaire surveyed villagers’ basic information (e.g., household number, gender). The subsequent sections included questions on (a) types and frequencies of raw fish dishes consumed, (b) consumption reasons, (c) *O. viverrini* awareness and prevention, and (d) raw fish dish sharing and fish procurement locations.

For the types and frequencies of raw fish dishes consumed, participants were asked if they have consumed any of the three *Isan* raw fish dishes, *koi pla*, *pla som* and *pla ra*, and their consumption frequencies (i.e., daily, weekly, monthly, or annual special occasions). *Koi pla*, or freshly made raw fish salad, is a mixture of finely chopped raw fish, lime juice, herbs and spices. *Pla som*, sticky rice-fermented fish or sour fish, is a lightly fermented fish dish, mixed with raw fish, salt, garlic and rice, with two to seven days of fermentation. *Pla ra*, a highly salted fish dish, has long-term fermentation from several months to one year, and is also often used as a seasoning ingredient. The risk of *O. viverrini* infection could be higher for consuming *kio pla* and *pla som* because the former is eaten soon after it is made and the fermentation time for the latter is not long enough to kill the parasites. Indeed, viable *O. viverrini* metacercariae have been detected in these two dishes [31]. Nevertheless, *pla ra* may still contain metacercariae depending on the fermentation time and the amount of salt used. Some *pla ra* sold in local markets have been detected with metacercariae despite the degeneration of their morphology [32].

For consumption reasons, five options based on prior studies (e.g., [19, 33, 34]) were compiled, including: Preference for taste/delicious; family/Isan tradition; social gathering; main source of meat/protein; and convenience. Participants could select more than one option, as there could be multiple reasons for consumption. For *O. viverrini* awareness and prevention, participants were asked about their sanitation practices (i.e., open defecation or not), awareness of the health consequence associated with raw fish consumption, and their willingness to avoid raw fish consumption should they be aware of the health consequence. For raw fish dish sharing and fish procurement locations, participants were surveyed for the households with which they shared the raw fish dishes and where they obtained fish from to make their raw fish dishes.

### Data analysis

#### Examining ecosystem disservices of parasitic infection

To assess if the village surrounding water bodies potentially provided ecosystem disservices of *O. viverrini* transmission, the following indicators were calculated. Snail species compositions were calculated as relative abundances of individual species in percentages. The proportions of the sampling plots with infected *Bithynia* snails and fecal contaminations were derived. Cerarial shedding of parasitic infection rates in snails and the means and ranges of fecal contamination in water samples were computed. Proportions of the participants who practiced open defection were analyzed from the questionnaire survey. Then, the two-sample *t*-test was used to assess the statistical difference between all quantitative indicators of the two villages, unless otherwise specified. All statistical analyses of this study were performed using the statistical software R 3.6.3 on R Studio [35], with the statistical significance level set at *P* < 0.05.

#### Investigating consumption habits and their relations to cultural and provisioning ecosystem services

To investigate the linkages between raw fish consumption and ecosystem services, participants’ questionnaire responses on preference for taste/delicious, family/Isan tradition and social gathering were grouped as relevance to cultural ecosystem services, and the percentage of these responses was computed. Likewise, participants’ responses on main source of meat/protein and convenience were grouped as provisioning ecosystem services.

The consumption frequencies of different dishes were first computed to the average days per year for comparison. Then, comparison was made between male and female because gender has been considered as a major demographic variable influencing food consumption behavior [36]. Prior study has suggested that males like to eat raw fish dishes particularly *koi pla* [33], as it embodies strength and power, which are traditionally associated with masculinity [19]. Univariate linear regression analysis was then performed for each village following Eq. (2). In each regression model, gender was used as the independent variable (*X*) with female as the baseline gender and male as the variable gender. The consumption frequencies of higher risk dishes, i.e., *koi pla*, *pla som* and a combination of both, were individually used as the corresponding dependent variables (*Y*).

2 $$Y=\alpha +\beta X+\varepsilon,$$

where *a* denotes the intercept for the baseline gender, *β* denotes the regression coefficient for the variable gender, and *ε* denotes the error term for each model. In addition, the gendered differences of reasons for raw fish consumption were analyzed following Eq. (2) to investigate the potential influences of specific motivation on any particular raw fish dishes. In each regression model, gender was again used as the independent (*X*) variable with female as the baseline gender and male as the variable gender, while the five reasons for consumption was used as the dependent variable (*Y*). Likewise, α denotes the intercept for the baseline gender, β denotes the regression coefficient for the variable gender, and ε denotes the error term for each model.

To understand the potential influences various perceptual and behavioral factors had on consumption habits, three sets of multivariate linear mixed effect regressions were performed for each village following Eq. (3):

3 $$Y=\beta X+\mu Z+\varepsilon,$$

where *Y* is the dependent variable representing consumption frequency (of combinations of *koi pla*, *pla som* and *pla ra*), willingness to avoid consumption (model ran with binomial distribution) and *O. viverrini* infection status (model ran with binomial distribution), respectively in each of the three sets of analyses. *β* represents the regression coefficient for *X*, the fixed effects vector comprising independent variables, including consumption frequency, two consumption reasons of ecosystem services (i.e., cultural and provisioning), three perceptual and behavioral factors (i.e., awareness of health consequence, willingness to avoid consumption, and participation in food sharing activities), and gender. The variable for consumption frequency was removed as a fixed effect variable when it was used as the dependent variable, and the perceptual factor of willingness to avoid consumption was removed as a fixed effect variable when it was used as a dependent variable. To account for the variation in characteristics between village BN and BT, a random intercept of *Z* was included in the models with their corresponding coefficient of *µ*. Lastly, *ε* represents the error term for the model.

Data on *O. viverrini* infection status used in Eq. (3) were obtained from the local health center that coordinated *O. viverrini* prevalence survey with the Cholangiocarcinoma Screening and Care Program (CASCAP) from 2016 to 2019. The overall human infection prevalence was calculated first for the two villages. *O. viverrini* infection status of individual villagers who participated in the questionnaire survey of this study was then extracted from the CASCAP data for the analyses of this study.

#### Analyzing raw fish dish sharing networks, fish procurement locations and *O. viverrini* infection risk

Participants’ responses on the households with which they shared the dishes, and where they obtained fish from to make their raw fish dishes were analyzed to assess if villages had varying networks of sharing and different environmental connections for fish procurement. Because prior study suggested that greater connectivity among households might increase human *O. viverrini* infection risk [18], network analysis was conducted to assess the potential association between household connections through raw fish dish sharing and *O. viverrini* infection risk. The graph theory approach was used to show the relative positions and relationships between individual entities and their connections in a network [37], with the entities and the relationships among them respectively conceptualized as a set of nodes and links. Accordingly, nodes were used to represent households that reported raw fish sharing behavior and the fish procurement locations. Links were used to represent the connection between two households that shared raw fish dishes and between the households and their fish procurement locations. No physical distances were used as the analyses were based on household connections of fish dish sharing activities or fish procurement locations.

Two sets of network graphs were created using the igraph R package [38]. The first set of the graphs focused on comparing the degree of raw fish dish sharing between BN and BT households, and the following indices were quantified: number of nodes, number of components, network density, mean degree centrality, and mean betweenness centrality. A component is a group of connected nodes; a higher number of components suggests more groups of sharing activities in individual village. Network density is computed as the proportion of the number of links to all possible links in the given network, illustrating the interconnectedness of the sharing activities. Degree centrality of a node measures the number of other distinct nodes connected to it by a link; hence, the higher the degree centrality, the more prominent the node is in the network because of its potential to directly interact with more entities. Alternatively, betweenness centrality measures the frequency of which a node sits on the shortest path between a pair of nodes, indicating the effectiveness of the node in bridging nodes in a network. As degree centrality is indicative of the level of social connectivity of a household in its food sharing network, degree centrality calculated from the first set of networks without accounting for connections to fish procurement sites, were used as one of the fixed effect independent variables in aforementioned the linear mixed effect models with Eq. (3).

The second set of the graphs incorporated fish procurement locations and their connections to households into the first set. For both sets of graphs, household nodes with *O. viverrini* positive infection, negative infection, or not tested were visualized. The probable influence of connections to fish procurement locations and sharing activities on *O. viverrini* infection risk was assessed.

## Results

### Ecosystem disservices of parasitic transmission

A total of 33 and 31 plots respectively from the surrounding water bodies of BN and BT were sampled for snails and *E. coli* testing. Live snails of 1005 and 961 respectively were collected for BN and BT for species composition analysis. BN had six snail taxa identified, more diverse than 3 taxa found in BT. For both villages, the majority of the snail species collected were *Bithynia* spp. snails (95.9% for BN, 98.3% for BT); *Pomacea* spp. was the second dominant snail species but it only took up 1.3–1.4% of the composition (Additional file 1: Table S1).

Cercarial shedding showed that close to half (48.5%) of the BN plots reported infected *Bithynia* snails, while slightly less than one-third of the BT plots (29.0%) had infected snails. No *O. viverrini* infection, however, was detected in the *Bithynia* snails sampled. Nonetheless, the snails could still carry other types of parasites and their cercariae were found in the study area. More types of parasite cercariae were detected in BN surroundings, while *Echinostome* and *Xiphidiocercaria* were found in both BN and BT (Table 1).

Examination of water samples revealed that 71.0% of the BN plots were contaminated with fecal contents, compared to 51.6% of the BT plots. BN plots also had a higher mean *E. coli* content at 339 CFUs/100 ml and a wider range from 0 to 5300 CFUs/100 ml than those at BT (Table 1). No visible spatial pattern of high CFU aggregations was observed in both village surroundings, but a statistically significant correlation was detected between the CFU counts and *Bithynia* snail parasitic infection rates in the BN plots (*P* = 0.000). Questionnaire survey on sanitation practice suggested that about 16% of the participants (15.6% for BN and 16.1% for BT) from the study area viewed open defecation as a common practice (Table 1). As the difference between the two villages is not statistically significant, open defecation practice was unlikely to directly contribute to the fecal content variation in the water bodies surrounding the two villages.

**Table 1:** Indications of ecosystem disservices of the water bodies surrounding BN and BT villages

Indications of ecosystem disservices	BN	BT
Type of cercariae and their infection rate in snails (%)		
* Opisthorchis viverrini*	0.0	0.0
Echinostome cercaria	0.1	0.1
Furcocercous cercaria	0.1	0.0
Monostome cercaria	0.2	0.0
Mutabile cercariae	0.1	0.0
Ophthalmoxiphidiocercariae	0.1	0.0
Pleurolophocercous cercaria	0.0	0.1
Xiphidiocercaria	3.5	1.4
Sample plots with fecal contamination (%)	71.0	51.6
Fecal contamination in water (CFUs/100 ml)		
Mean	339	125
Range	0–5300	0–1080
Open defection by villagers (%)	15.6	16.1

*BN* *Ban* Nam, *BT* *Ban* Tong

### Consumption habits and their relations to cultural and provisioning ecosystem services

#### Frequencies of consumption

Sixty-four households from BN and 56 households from BT were interviewed for the questionnaire. The findings showed that 71.9% of the BN participants and 87.5% of the BT participants consumed raw fish dishes of various types at least once a year. *Pla ra* was the most frequently consumed dish in both villages, with BT (267.2 days/year) having a significantly higher consumption than BN (45.5 days/year) (*P* = 0.000) (Table 2). Villagers did not consume *koi pla* and *pla som* as often as *pla ra*. Nevertheless, BN exhibited a higher frequency of *koi pla* and *pla som* consumption combined, an average of 16.2 days, than that of 11.5 days in BT, although the difference was not statistically significant.

**Table 2:** Frequencies of raw fish consumption in the surveyed villages BN and BT

Consumption frequency (average days/year)	BN	BT
*Koi pla*	7.5	8.2
*Pla som*	8.6	3.3
Pla ra*	45.5	267.2
*Koi pla* and *pla som* combined	16.2	11.5
All three combined*	61.6	278.7

*BN* *Ban* Nam, *BT* *Ban* Tong; *P < 0.05

Analysis of gender consumption showed significant differences in most raw fish dishes of higher risk (Table 3). Both BN and BT male respondents were likely to consume *koi pla* much more frequently than female respondents. On average, BN males would consume *koi pla* 32.4 days/year, compared to 1.5 days/year for BN females (*P* = 0.025). The difference was much more for BT, where males would likely consume *kio pla* 41.2 days/year, compared to 0.8 days/year for females (*P* = 0.015). When the consumption of higher risk dishes of *koi pla* and *pla som* was combined, both villages exhibited more frequent consumptions by males than females. Particularly in BN, male consumption frequency could go beyond two months, averaged at 64.5 days/year, compared to 4.1 days/year for female (P = 0.026).

**Table 3:** Gender differences in consumption frequency of various raw fish dishes

Village	Dish type	Intercept	Estimate
BN	Koi pla*	1.5 ± 6.1	32.4 ± 14.1
	Pla som*	2.6 ± 6.2	32.2 ± 14.2
	*Koi pla* + pla som*	4.1 ± 12.2	64.5 ± 28.3
BT	Koi pla*	0.8 ± 6.9	41.2 ± 16.3
	*Pla som*	3.5 ± 0.8	-0.8 ± 1.8
	*Koi pla* + pla som*	4.3 ± 6.9	40.4 ± 16.3

Intercept denotes the mean consumption frequency of the baseline gender (female); estimate shows the mean consumption frequency of the variable gender (male) relative to the baseline gender. The standard error for each intercept and estimate is included following the ± symbol. *BN* Ban Nam, *BT* Ban Tong. * *P* < 0.05

#### Cultural and provisioning ecosystem services of raw fish consumption

More than 85% of the participants claimed that they were aware of the health consequences of raw fish consumption (Table 4). Despite this, less participants, 68.8% in BN and 57.1% in BT, were willing to avoid the consumption. Both villages substantially relied on the cultural ecosystem services, marked by 82.8% of the BN participants and 94.6% of the BT participants mentioning preference for taste, Isan culture, or social gathering as their reasons for consumption (Table 4). Furthermore, BT participants (76.8%) preferred the raw fish tastes more than BN participants (57.8%), while a much higher proportion of the participants in BN (42.2%) than in BT (12.5%) consumed raw fish due to social gathering (*P* = 0.000). Close to two-thirds of the participants from both villages consumed raw fish because of the traditional Isan culture.

In contrast, reasons for consumption associated with provisioning ecosystem services were less chosen by the participants, with more BN participants relying on such provisioning ecosystem services than BT (Table 4). This was evident in that more than double of the BN participants (29.7%) consumed raw fish dishes for convenience than BT (14.3%). There was still about one-tenth (9.4%) of the BN participants took raw fish dishes as their main source of protein, but only 1.8% of the BT participants consumed raw fish as the main protein source.

**Table 4:** Consumption habits, knowledge and perception towards raw fish dishes of BN and BT villagers

Factors	BN (%)	BT (%)
Aware of health consequences	89.1	85.7
Willingness to avoid raw fish	68.8	57.1
Reasons for consumption		
Cultural ecosystem services*	82.8	94.6
Preference for taste*	57.8	76.8
Isan culture	67.2	64.3
Social gathering*	42.2	12.5
Provisioning ecosystem services	29.7	16.1
Convenience*	29.7	14.3
Main source of protein	9.4	1.8

*BN* *Ban* Nam, *BT* *Ban* Tong; *P < 0.05

Results of the gendered difference in consumption reasons generally showed similar patterns in both BN and BT (Table 5). For BN villagers who consumed raw fish for *Isan* culture, social gathering and convenience, the odds of it being a male was higher than the odds of it being a female. For BT villagers, the higher odds of being a male was also observed in consumption for social gathering and convenience. Statistical significance was detected in BT for gendered difference in consumption for social gathering (*P* = 0.001). The odds of a BT villager consuming raw fish dishes for social gathering reason being a male was 22 times higher than the odds of it being a female.

**Table 5:** Gender differences in reasons for consumption of various raw fish dishes in BN and BT villages

Variable	BN		BT	
	Intercept	Estimate (gender male)	Intercept	Estimate (gender male)
Preference for taste	0.39 ± 0.28	− 0.39 ± 0.64	1.41 ± 0.37	− 1.01 ± 0.74
Isan culture	0.64 ± 0.29	0.46 ± 0.73	0.83 ± 0.32	− 1.23 ± 0.72
Social gathering	− 0.47 ± 0.29	0.81 ± 0.65	− 3.09 ± 0.72	3.09 ± 0.96 (odds ratio = 22)*
Convenience	− 1 ± 0.31	0.66 ± 0.66	− 1.9 ± 0.44	0.51 ± 0.9
Protein	− 2.24 ± 0.47	− 0.16 ± 1.15	− 3.81 ± 1.01	− 16.76 ± 5606.84

Intercept denotes the log of the odds of the baseline gender (female) consuming raw fish for the particular reason; estimate shows log of the odds of the variable gender (male) consuming raw fish for the particular reason relative to the baseline gender. The standard error for each intercept and estimate is included following the ± symbol. Odds ratio was included for the relationship that was statistically significant. *BN* *Ban* Nam; *BT* *Ban* Tong. *P < 0.05

Analysis of the potential influences various perceptual and behavioral factors had on consumption habits (Table 6) showed that consumption frequency was positively associated with participation in food sharing activities (*P* = 0.003). For all the respondents from both villages, those who indicated ‘yes’ for participating in food sharing activities were likely to consume raw fish dishes 86.8 days/year more than those who indicated ‘no’. In another version of the model where five reasons for raw fish dish consumption were analyzed (Additional file 1: Table S2), participation in food sharing activities again showed significantly positive correlation with consumption frequency. Conversely, degree centrality was significantly negatively correlated to consumption frequency (*P* = 0.037), with an increase in one more food sharing connection leading to consumption frequency of 35.9 fewer days/year (Table 6).

As for the associations between willingness to avoid consumption and various factors analyzed, statistical significance was also detected for participation in food sharing activities with reasons for consumption categorized into ecosystem services (Table 6). Among the villagers expressing willingness to avoid raw fish, the odds of one participating in food sharing is 19% of the odds of one who did not (*P* = 0.000). In other words, the odds of a villager expressing willingness to avoid raw fish without participation in food sharing is 5.3 times more than one who expresses willingness to avoid raw fish but have participated in food sharing. Analysis of CASCAP *O. viverrini* survey data that recruited more villagers revealed that overall human *O. viverrini* infection prevalence between 2016 and 2019 was higher in BN (49.3%) than in BT (25.0%). When the CASCAP data were analyzed only for the villagers who participated in the questionnaire survey of this study, lower human *O. viverrini* prevalence was found for both villages, 17.2% in BN and 3.6% in BT. Investigation of the associations between participant’s *O. viverrini* infection status and the behavioral and perceptual variables, however, showed that none of the variables exhibited statistical significance (Table 6).

**Table 6:** Potential influences of perceptual and behavioral factors, with reasons for consumption categorized into ecosystem services, on consumption habits of BN and BT villagers

Variable	Consumption frequency	Willingness to avoid consumption	*O. viverrini* infection status
	Estimate	Estimate	Estimate
Cultural ecosystem services	6.96 ± 46.98	0.15 ± 0.85	11.79 ± 1170.45
Provisioning ecosystem services	42.62 ± 27.51	0.13 ± 0.42	0.75 ± 0.55
Willingness to avoid consumption	− 7.48 ± 27.47	–	1.25 ± 0.83
Awareness of health consequences	− 27.66 ± 83.83	0.92 ± 1.2	11.36 ± 1002.42
Participation in food sharing activities	86.81 ± 28.86*	− 1.67 ± 0.48* (odds ratio = 0.19)	1.06 ± 0.68
Degree centrality of food sharing	− 35.91 ± 16.97*	0.38 ± 0.25	0.06 ± 0.28
Frequency of consumption	–	0 ± 0	0 ± 0
Gender: male	27.32 ± 40.39	− 0.3 ± 0.59	0.14 ± 0.9

Consumption frequency showed all three raw fish dishes consumed. The standard error for each intercept and estimate is included following the ± symbol. The symbol ‘–’ denotes no result because the specific variable is removed for regression when that variable is used as the dependent variable. Odds ratio for the analysis of willingness to avoid consumption and *O. viverrini* infection status was included for the relation that was statistically significant. *BN* *Ban* Nam; *BT* *Ban* Tong. *P < 0.05

### Raw fish dish sharing networks, fish procurement locations and *O. viverrini* infection risk

**Fig. 2:**
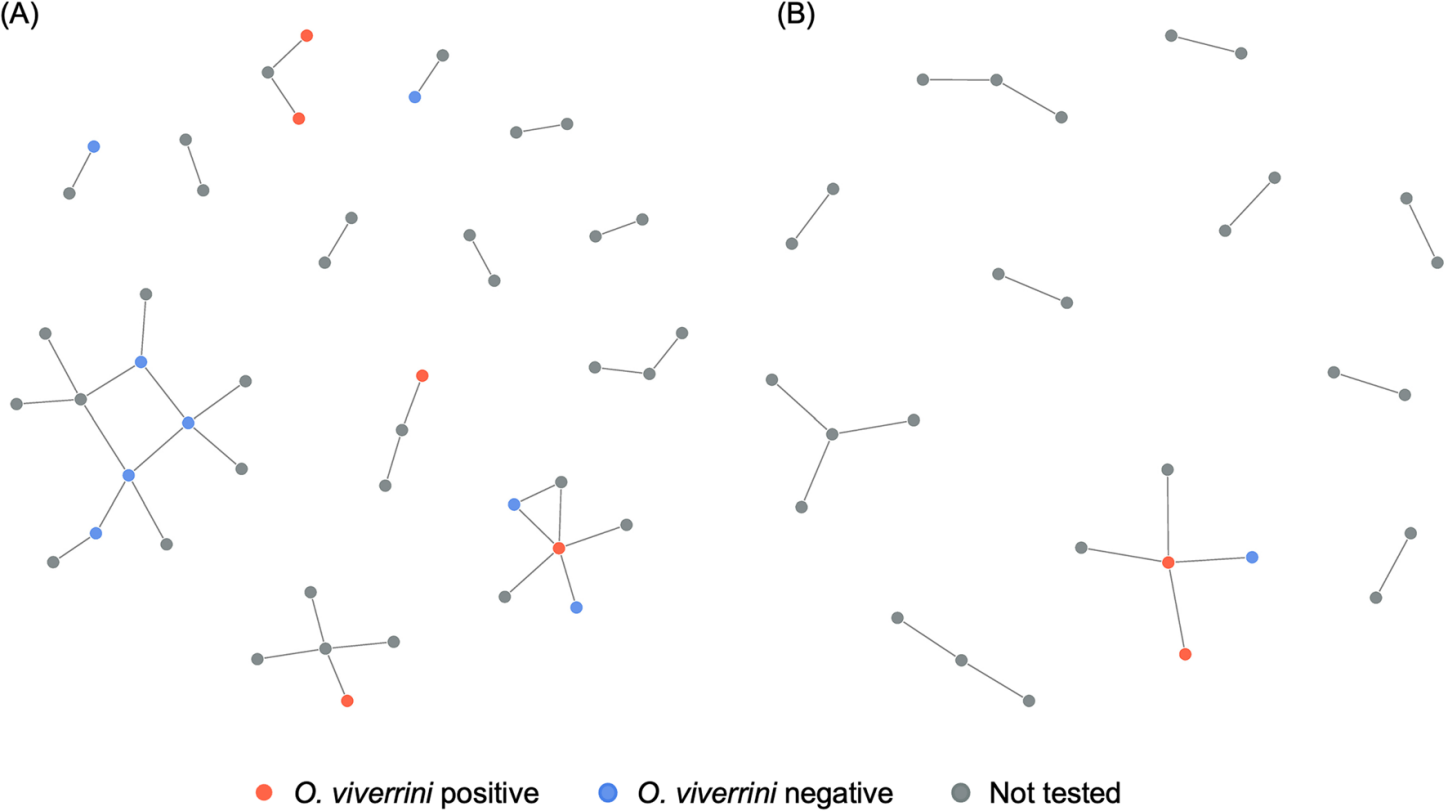
Social networks of (**A**) BN and (**B**) BT villagers who shared raw fish dishes beyond their own household. Household numbers were not shown for confidentiality. *BN* *Ban* Nam, *BT* *Ban* Tong

Raw fish dish sharing beyond one’s own household remained common, marked by 62.5% of BN and 51.8% of BT participants. BN had 13 components while BT had 11; several BN households also had more extensive sharing activities with at least four other households in the same village compared to BT (Fig. 2). Moreover, network indices revealed noticeable higher values in mean degree centrality and mean betweenness centrality for BN (Table 7). The mean degree centrality for BN ranged from 1.61 to 1.87, compared to the range from 1.29 to 1.33 for BT; BN also had higher mean betweenness centrality values ranging from 0.93 to 2.59, compared to the range from 0.17 to 0.47 for BN (Table 7). These measurements signified that more BN households than BT had the potential of directly joining food sharing activities with other households in the same village.

**Table 7:** Indices of food sharing network in the surveyed villages BN and BT

Network indices	Raw fish dish type	BN	BT
Number of nodes	All raw fish dishes	46	29
	*Koi pla*	28	17
	*Pla som*	38	12
	*Pla ra*	41	17
Number of components	All raw fish dishes	13	11
	*Koi pla*	9	6
	*Pla som*	11	5
	*Pla ra*	13	7
Network density	All raw fish dishes	0.0415	0.0470
	*Koi pla*	0.0608	0.0809
	*Pla som*	0.0498	0.121
	*Pla ra*	0.0402	0.0809
Mean degree centrality	All raw fish dishes	1.87	1.31
	*Koi pla*	1.64	1.29
	*Pla som*	1.84	1.33
	*Pla ra*	1.61	1.29
Mean betweenness centrality	All raw fish dishes	2.59	0.379
	*Koi pla*	0.930	0.471
	*Pla som*	1.61	0.167
	*Pla ra*	1.17	0.235

*BN* *Ban* Nam, *BT* *Ban* Tong

Villagers obtained fish from multiple sources to make raw fish dishes, not only relying on their surrounding physical environments (e.g., rivers), but also markets and mobile sellers (Table 8). Comparison of fish procurement locations revealed several statistical differences between the two villages (Table 8). Most of the BN villagers (65.6%) obtained their fish from rivers to make *koi pla*, *pla som*, or *pla ra*, where only a small portion of the BT villagers (5.4%) procured their fish from. Conversely, market was the most common place for BT villagers to obtain their fish (66.1%). Although there was no reservoir present in the study area, some participants mentioned about getting their fish from the reservoirs should they travel further to the Lam Pao reservoir in the northern Kalasin.

**Table 8:** Fish procurement locations mentioned by BN and BT participants

Location	BN (%)	BT (%)
Pond*	18.8	37.5
River*	65.6	5.4
Rice paddy*	0	37.5
Market*	28.1	66.1
Mobile seller	7.8	16.1
Neighbor	15.6	7.1
Reservoir*	14.1	1.8

*BN* *Ban* Nam; *BT* *Ban* Tong; *Statistically different between two villages at *P* < 0.05

**Fig. 3:**
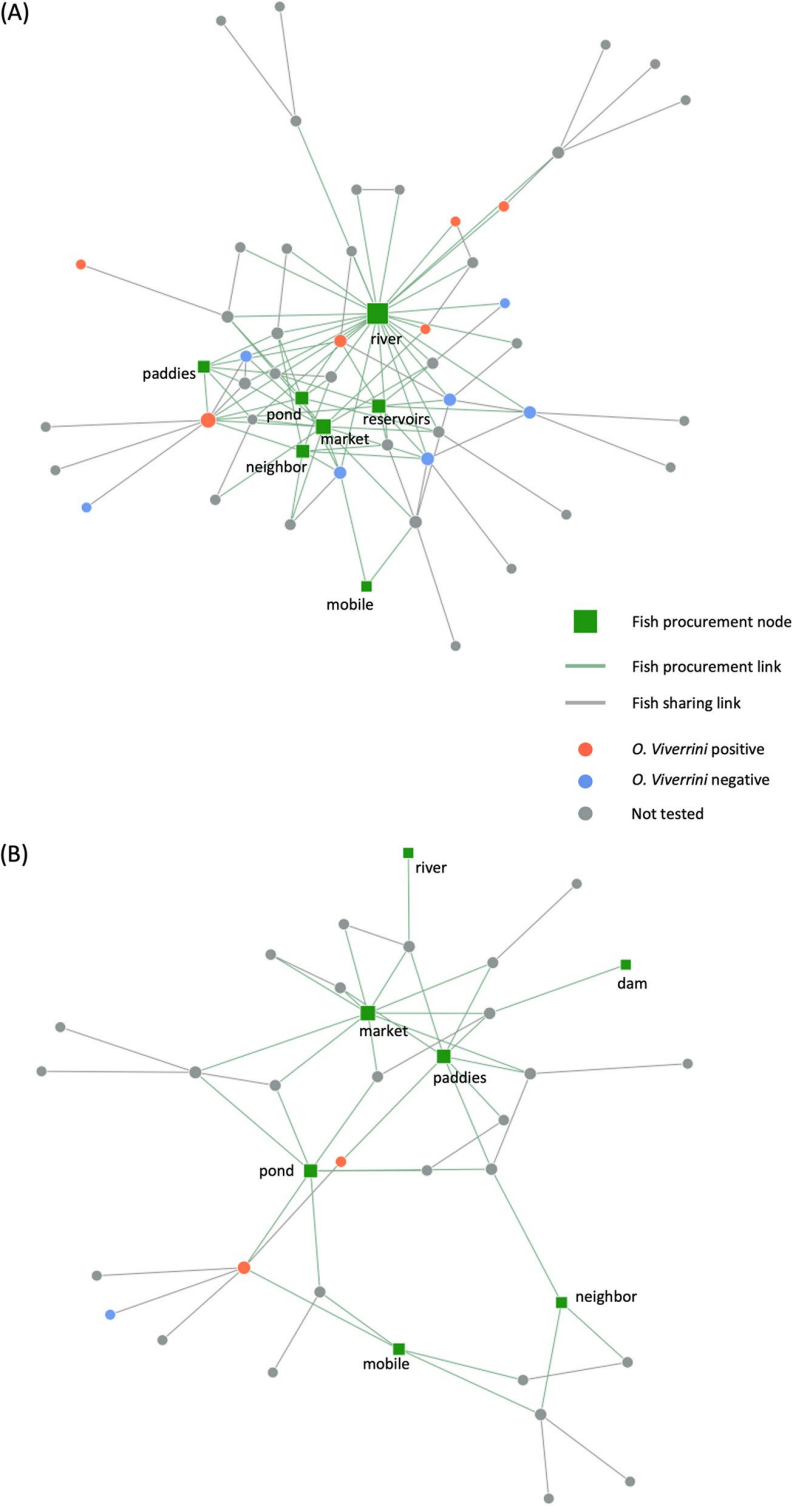
Network graphs of raw fish dish sharing, connections to fish procurement locations, and *Opisthorchis viverrini* infection status among (**A**) BN and (**B**) BT villages. Household node sizes were scaled based on their sharing connections; fish procurement node sizes were scaled according to the fish procurement connections. The scaling was done together for both BN and BT to allow direct comparison. *BN* *Ban* Nam, *BT* *Ban* Tong

The network graphs incorporating fish procurement locations extensively joined the food sharing components together. BN consistently exhibited a more extensive network than BT (Fig. 3). River appeared as the most prominent network hub in BN. Some BN households had three or four different fish procurement locations, and quite a few BN households with raw fish dish sharing practice also obtained their fish from other sources beyond the river. This was illustrated by the multiple links between individual households and various fish procurement locations, particularly ponds, reservoir, market and paddies. As for BT, market, paddies and ponds served as the network hubs connecting villagers’ raw fish practices, and most BT households obtained their fish from just one or two locations. Five *O. viverrini* positive households were in the BN network graph, most of which were directly connected to the river fish source. Alternatively, two *O. viverrini* positive households were in the BT graph and they were directly linked to different procurement sources, including paddies, pond, and mobile seller. Analysis of the infection status against sharing degree centrality showed a statistical significance for BT where on average, villagers with *O. viverrini* positive records had more sharing connections than those without *O. viverrini* positive records (*P* = 0.021). The association, however, was not statistically different for BN.

## Discussion

### Ecosystem disservices of parasitic transmission

The transmission from the definitive human host to the first intermediate *Bithynia* snail host is the phase where *O. viverrini* is introduced into the freshwater environment [29]. Although prior studies on *Bithynia* snails have found low *O. viverrini* infection rates, usually less than 1% [39], just one infected snail can release hundreds of cercariae in the water environment, triggering exponentially high infection rates in the second intermediate freshwater fish hosts and subsequently endangering human health. Hence, *Bithynia* snails can be considered as the amplification point of *O. viverrini* transmission [25]. The dominance of *Bithynia* snails, at over 95% of the snail species composition in both villages, suggested that the freshwater environments of the study area have the potential for providing ecosystem disservices of parasitic transmission.

Although no *Bithynia* snail was tested *O. viverrini* positive in the study area, infections by other parasites were observed. In particular, the presence of *Echinostome* cercariae in the *Bithynia* snails from both villages confirmed the on-going process of human to snail transmission because, similar to *O. viverrini*, the eggs of the *Echinostome* parasite were also passed through human feces into snail habitats [40]. The high relative abundance of *Bithynia* snails and the prevalence of other cercariae thus indicate that *Bithynia* snails found in both villages are still susceptible to *O. viverrini* infection, which can potentially contribute to the persistence of *O. viverrini* infection in the area. Water quality analysis of fecal contents further showed that BN could be of higher susceptibility to ecosystem disservices of parasitic transmission, because the village had more plots with fecal contamination and a higher mean of CFU counts than BT (Table 1). It should, however, be noted that, despite the proven technology of the Coliscan method for estimating fecal contents, the method could not distinguish whether the detected fecal matter was of human or animal origin. Hence, it was possible that the fecal matters in the village surroundings were from other *O. viverrini* reservoir hosts, such as cats and dogs, or other domestic animals, such as water buffalo, kept by the villagers. Nevertheless, the significant association between *Bithynia* snail infection rates and the CFU counts measured in the BN plots also suggested that in the event of *O. viverrini* egg-loaded fecal contamination in the study area, the BN plots would likely to be the point of *Bithynia* snail infection that could give rise to *O. viverrini* proliferation.

### Derivation of ecosystem services through raw fish consumption

Villagers have their consumption habits driven heavily by derivation of cultural ecosystem services, and still derive provisioning services from the surrounding environments by obtaining freshwater fish as their main source of protein. Gender differences in consumption is also detected, with males consuming higher risk dishes of *koi pla* and *pla som* significantly more often than females (Table 3 and S3). Prior studies have found gender difference in foodborne parasitic infections [41]. This suggests a gendered variation in infection risk exposure, which could be attributed to the differences in diet and consumption habits [19]. The statistical examination of gender against raw fish dish consumption frequencies in this study thus provides further quantitative understanding to support this notion, showing that in some villages like BN, male consumption frequency could be up to two months more a year than female.

The deeply imbedded raw food culture in the *Isan* community has been underscored as the main driver for persistent raw fish consumption [33]. However, with globalization and urbanization, food choices for the rural communities are expanding; other convenient sources of protein from fast food and instant food are becoming more accessible and common in daily household diets [34, 42]. Traditional raw fish dishes may no longer be the main protein source and are consumed more so for cultural and social functions. This is evident in both villages, where the presence of convenience stores and eateries widens villagers’ food choices. Nevertheless, more than 70% and 85% of the BN and BT participants respectively reported their continuous raw fish dish consumption at least once a year. The proportion of people who consume for the cultural ecosystem services provided also far exceeds that for the provisioning services provided (Table 4). In addition, there are two statistically significant associations that further highlight the shift in consumption tendency from provisioning reasons to cultural motivations (Table 6). For both villages, consumption due to participation in food sharing activities significantly reduces the odds of an individual being willing to avoid raw fish consumption, which supports the importance of cultural ecosystem services. Participation in food sharing activities is also correlated to a higher frequency of consuming all raw fish dishes, supporting the notion that cultural practice of raw fish consumption is rooted in social connection. Alternatively, raw fish consumption for protein intake significantly encourages higher consumption frequencies of *kio pla* and *pla som* (Additional file 1: Table S3), the higher risk dishes. There still exists an intrinsic behavior of raw fish dish consumption that is resilient to external urban influences on local food variety and choices. This may motivate villagers to continuously carry out small scale fishing activities to procure raw fish from their surrounding environments. Consequently, food safety and villagers’ wellbeing are still of concern due to the potential risk of *O. viverrini* infection that comes along with deriving ecosystem services.

Nevertheless, while raw fish consumption motivated by cultural and social functions remains, there are observable trends in perceptual corrections and behavioral improvements that could abate the infection risks associated with persistent consumption habits. Firstly, willingness to avoid raw fish consumption leads to a lower consumption frequency of all raw fish dishes combined, suggesting that understanding proper food safety practices can lead to reduced risks associated with the consumption. Furthermore, the negative correlation between degree centrality of food sharing and consumption frequency suggests that *O. viverrini* infection risk is unlikely to amplify with increased social connection through food (Table 6). This is contrasting to findings from prior study where consumption frequency and diversity are both positively correlated with higher degree of food sharing, leading to a higher infection risk [18]. The findings from this study thus suggests that gradual adjustments to raw fish consumption habits among villagers could help to achieve a balance between the preservation of raw fish consumption cultural identity, but mitigate the prospect of developing related adverse health consequences.

### Interactions between geographic settings of villages, fish procurement and sharing networks, and infection risk

Besides the derivation of cultural and provisioning ecosystem services, the geographical setting of a village and a villager’s choice of fish procurement location further reflect the direct interaction villagers have with their environments. In this study, BN is in close proximity to a river, and its villagers mostly fish from the river, followed by purchasing fish from the market (Table 8; Fig. 3A). In contrast, BT is located further inland with no large water bodies nearby to fish from (Fig. 1) and the village is also closer to the Nong Paen sub-subdistrict where a larger food market is available. BT villagers thus mostly obtain fish from the market, followed by small ponds and rice paddies (Table 8; Fig. 3B). Therefore, village locations and their surrounding environments affect the availability of fish procurement locations, potentially contributing to the significantly higher proportion of BN villagers fishing from the river and that of BT villagers obtaining fish from the market. Subsequently, fish procurement locations with high potential to harbor *O. viverrini* infected fish may increase the human infection risk. Prior work on another liver fluke species in Vietnam reports a significantly higher infection rate in people who eat raw fish caught from a nearby river than those who consume fish taken from farmed ponds [41]. The finding of this study echoes prior work, as BN, whose villagers mostly obtain fish from the nearby river, also has a higher overall human prevalence of infection at 49.3% than BT at 25.0% [23]. When the prevalence of infection is computed based on only the participants recruited for this study, BN participants still have a much higher *O. viverrini* prevalence at 17.2%, compared to BT at 3.6%. The geographic settings of the villages might have exposed their villagers to different levels of infection risks.

With the diversification of rural economy, alternate sources of fish from markets and mobile sellers are available to villagers, without the need to actively interacting with the natural environment to obtain fish. It has been reported that larger, more economic fish species, which are also the species less susceptible to *O. viverrini* infection, are often transported to economically active towns for sale while markets in local villages receive the smaller, less economic fish, which are more susceptible to *O. viverrini* infection [34]. Furthermore, since reservoirs harbor richer fish population, it is likely that the source of fish to the markets are also from the reservoirs [43]. Impounded water bodies like reservoirs are known to have even higher *O. viverrini* fish infection rates as compared to rivers for all seasons [43, 44], which further increases the risk of local villagers receiving infected fish in their purchases.

In addition to individual’s consumption habit and perception towards traditional raw fish dishes, social interactions in the community may affect an individual’s exposure to disease risk [42, 45, 46]. The use of social network analysis illustrates the extents and connections of raw fish dish sharing, and how these interactions might potentially contribute to the varied human *O. viverrini* prevalence in the two villages. Three network characteristics of BN might have exposed its villagers to higher *O. viverrini* infection risk and cause more *O. viverrini* positive households (Table 7; Fig. 3). First, BN had larger component numbers than BT in all types of raw fish dishes shared (Table 7), signifying more groups of raw fish sharing activities within BN. Second, the mean degree centrality and mean betweenness centrality for all types of raw fish dishes were also all higher in BN than in BT (Table 7), indicating that more BN households directly share raw fish dishes with other households within the village. Third, quite a few of the BN households obtained fish from various fish procurement locations, while most of the BT households procure their fish from just one or two sources (Fig. 3).

It should, however, be also noted that there were less BT households being interviewed in the survey, possibility resulting in its slightly lower number of components than BN for all raw fish dishes combined (Table 7). Less *O. viverrini* positive households were also found in BT among the participants surveyed in this study, but statistical significance was detected for the infectious status against sharing degree centrality for BT, not for BN. This indicated that in BT, *O. viverrini* positive individuals could have more sharing connections than those without *O. viverrini* positive records.

### Implications for disease management

This study shows that raw fish consumption is still a common practice in the study area. Urbanization might have triggered villagers to transit from traditional *Isan* raw food culture to a more western food culture for processed and pre-packed food [4, 34]. However, exposure to *O. viverrini* infection is not completely eliminated as most villagers still consume raw fish dishes. The consumption frequencies of higher risk dishes of *koi pla* and *pla som* have also been found to be significantly associated with males and the provisioning ecosystem service of protein source for BN, and participation in food sharing activities for BT (Table 3 and S3). Although the majority of the participants claim that they are aware of the health consequences of raw fish consumption, there can be misconceptions from *O. viverrini* life cycle to food safety, as illustrated by [19] that various ingredients were believed and used to kill the parasite, as opposed to fully cook the fish. Hence, continuous health education and disease control strategy remain critical to ensure that the villagers are equipped with accurate information regarding *O. viverrini* prevention and the role of their environment in providing both ecosystem services and disservices to their livelihood.

Villagers’ strong reliance on raw fish for cultural ecosystem services (Table 4) makes it culturally insensitive to forcibly remove the raw fish consumption tradition entirely. Therefore, instead of discouraging the consumption of traditional raw fish dishes, the correct culinary treatment and fermentation procedures could be introduced, or reminded, to the *Isan* communities to effectively avoid the contact with viable *O. viverrini* metacercariae. Personal correspondence with local villagers exemplified this possibility as they shared that *pla som*, a dish that is made of lightly fermented fish, can be prepared with fully cooked fish. Indeed, the ‘Lawa model’, a liver fluke control program based on One Health approach, has been implemented in another *Isan* province to present ‘cooked’ raw fish recipes, among other novel health education methods [47]. Such alternatives should be made aware for the wider *Isan* community to adopt so that the potential adverse impacts from ecosystem disservices do not hinder the derivation of ecosystem services, particularly in practicing and preserving *Isan* tradition.

Another potential way to safeguard the food safety for villagers could be discouraging them from procuring fish from water bodies where the completion of the *O. viverrini* life cycle might persist. The reporting of open defecation, the presence of fecal contamination and the dominance of *Bithynia* snails in the water bodies of village surroundings (Table 1) show the potential for the *O. viverrini* life cycle to start, should the person who openly defecates be infected with the parasite. Rice fields and ponds that serve as shared habitats for both intermediate hosts, *Bithynia* snails and cyprinid fish, are of particular concern. If cyprinid fish were caught from these water bodies, then they are likely be contaminated with the parasite. Concurrently, education on sanitation practice should be continued and sewage treatment facilities should be strengthened, to prevent feces from getting into the freshwater environments.

### Limitations and future work

This study used fecal contamination in the village surrounding water bodies as a proxy for ecosystem disservices of *O. viverrini* transmission due to the low infection rate in *Bithynia* snail hosts. Despite the detection of other parasitic infections, no *O. viverrini* cercaria was found from *Bithynia* snail shedding. More sensitive diagnostic tools would be useful to provide a more accurate means of examining parasitic transmission in the water environments where the nonhuman part of the parasite life cycle occurs. For example, environmental DNA-based tools can be considered, as the method has demonstrated a higher sensitivity than conventional snail shedding in detecting parasitic transmission such as schistosomiasis [48]. Additionally, it would be desirable to conduct fish sampling from different procurement locations to investigate the *O. viverrini* infection level in fish and to trace the fish distribution network. Furthermore, the recruitment of the participants could target those that have been tested for *O. viverrini* infection to increase the sample sizes with *O. viverrini* infection status. A more complete *O. viverrini* prevalence survey data would be useful to improve the quantitative assessments between the characteristics of the sharing networks and *O. viverrini* infection risk.

## Conclusion

This study revealed that although the river-side village relied more on provisioning ecosystem services than the inland village to consume raw fish as their main source of protein, it was concluded that raw fish consumption habits of both villages were driven more by cultural motivations than the necessity of sustenance. Sharing of raw fish dishes beyond one’s own household remained common, and participation in raw fish dish sharing activities significantly reduced the odds of an individual being willing to avoid the consumption. The geographic settings of the villages potentially affected villagers’ fish procurement locations and infection risk, supported by the network analysis that the river-side village had more groups of raw fish sharing activities, more direct raw fish dish sharing interactions, and higher human prevalence of infection than the inland village. These findings highlighted the linkages between villagers and their surrounding ecosystem environments as pertinent determinants for foodborne parasitic disease risk.

## Supplementary Information

**Additional file 1:** **Table S1.** Snail species composition (%) in the surrounding water bodies of BN and BT villages. **Table S2. **Potential influences of perceptual and behavioral factors on consumption habits of BN and BT villagers. Consumption frequency was based on all three raw fish dishes consumed. Odds ratio for the analysis of willingness to avoid consumption and *O. viverrini* (*O.v.*) infection status was included for the relation that was statistically significant. **Table S3. **Potential influences of reasons of consumption (comparing five individual reasons vs. two reasons of ecosystem services), and perceptual and behavioral factors on consumption frequencies of BN and BT villagers. Consumption frequency was based on higher risk raw fish dishes, *koi pla* and *pla som*. **Table S4.** Potential influences of perceptual and behavioral factors, and network indices of degree centrality and betweenness centrality on consumption habits of BN and BT villagers. Consumption frequency wasbased on all three raw fish dishes consumed. **Table S5.** Potential influences of ecosystem services, perceptual and behavioral factors, and network indices of degree centrality and betweenness centrality on consumption habits of BN and BT villagers. Consumption frequency was based on all three raw fish dishes consumed.

## Data Availability

The datasets used and/or analysed during the current study are available from the corresponding author on reasonable request.

## Abbreviations

BN: *Ban* Nam
BT: *Ban* Tong
CASCAP: Cholangiocarcinoma Screening and Care Program
CFU: Colony forming unit

## References

[1] Woolhouse ME, Gowtage-Sequeria S (2005). Host range and emerging and reemerging pathogens. Emerg Infect Dis.

[2] Salyer SJ, Silver R, Simone K, Behravesh CB (2017). Prioritizing zoonoses for global health capacity building—themes from One Health zoonotic disease workshops in 7 countries, 2014–2016. Emerg Infect Dis..

[3] Adisasmito WB, Almuhairi S, Behravesh CB, Bilivogui P, Bukachi SA, One Health High-Level Expert Panel (2022). One health: a new definition for a sustainable and healthy future. PLoS Pathog.

[4] Sripa B, Tangkawattana S, Laha T, Kaewkes S, Mallory FF, Smith JF, Wilcox BA (2015). Toward integrated opisthorchiasis control in northeast Thailand: the Lawa project. Acta Trop.

[5] Oosterbroek B, de Kraker J, Huynen MM, Martens P (2016). Assessing ecosystem impacts on health: a tool review. Ecosyst Serv.

[6] Harrison S, Kivuti-Bitok L, Macmillan A, Priest P (2019). EcoHealth and One Health: a theory-focused review in response to calls for convergence. Environ Int.

[7] Vaz AS, Kueffer C, Kull CA, Richardson DM, Vicente JR, Kühn I, Schröter M, Hauck J, Bonn A, Honrado JP (2017). Integrating ecosystem services and disservices: insights from plant invasions. Ecosyst Serv.

[8] Reid W et al. Millennium Ecosystem Assessment, WRI: World Resources Institute. 2005. https://policycommons.net/artifacts/1360846/millennium-ecosystem-assessment/1974825/. CID: 20.500.12592/5qwvp6. Accessed 11 Sep 2022.

[9] Lyytimäki J, Sipilä M (2009). Hopping on one leg—the challenge of ecosystem disservices for urban green management. Urban For Urban Green.

[10] Dunn RR (2010). Global mapping of ecosystem disservices: the unspoken reality that nature sometimes kills us: ecosystem disservices. Biotropica.

[11] Patz JA, Confalonieri EC, Amerasinghe FP, Chua KB, daszak P, Hyatt AD, Hassan R, Scholes R, Ash N (2005). Human health: ecosystem regulation of infectious diseases. Ecosystems and Human Well-being: current state and Trends.

[12] Surtees G, Stanley NF, Alpers MP (1975). Mosquitoes, arboviruses and vertebrates. Man-made lakes and Human Health.

[13] Jobin W (1999). Dams and disease: Ecological Design and Health Impacts of large dams, canals and Irrigation Systems.

[14] Okamura B, Feist S (2011). Emerging diseases in freshwater systems. Freshw Biol.

[15] Sokolow SH, Jones IJ, Jocque M, La D, Cords O, Knight A (2017). Nearly 400 million people are at higher risk of schistosomiasis because dams block the migration of snail-eating river prawns. Philos Trans R Soc Lond B Biol Sci.

[16] Pawaputanon N, Mahasarakarm O. An Introduction to the Mekong Fisheries of Thailand. Mekong Development Series No. 5 Mekong River Commission, Vientiane, Lao PDR. 54pp. Available at: https://www.mrcmekong.org/assets/Publications/report-management-develop/Mek-Dev-No5-Mekong-Fisheries-Thailand-Eng.pdf. 2007.

[17] Gurven M (2004). To give and to give not: the behavioral ecology of human food transfers. Behav Brain Sci.

[18] Saenna P, Hurst C, Echaubard P, Wilcox BA, Sripa B (2017). Fish sharing as a risk factor for *Opisthorchis viverrini* infection: evidence from two villages in north-eastern Thailand. Infect Dis Poverty.

[19] Wang Y-C, Grundy-Warr C, Namsanor J, Kenney-Lazar M, Tang CJY, Goh LYW (2021). Masculinity and misinformation: social dynamics of liver fluke infection risk in Thailand. Parasitol Int.

[20] Wang Y-C, Feng C-C, Sithithaworn P, Feng Y, Petney TN (2011). How do snails meet fish? Landscape perspective needed to study parasite prevalence. EcoHealth.

[21] Trostle JA, Hubbard A, Scott J, Cevallos W, Bates SJ, Eisenberg JNS (2008). Raising the level of analysis of food-borne outbreaks: food-sharing networks in rural coastal Ecuador. Epidemiology.

[22] Alsaleh M, Leftley Z, Barbera TA, Sithithaworn P, Khuntikeo N, Loilome W (2019). Cholangiocarcinoma: a guide for the nonspecialist. Int J Gen Med.

[23] Cholangiocarcinoma Screening and Care Program. 2019. Isan Cohort https://cloud.cascap.in.th/. Accessed 8 Mar 2019.

[24] Brockelman WY, Upatham ES, Viyanant V, Ardsungnoen S, Chantanawat R (1986). Field studies on the transmission of the human liver fluke, *Opisthorchis viverrini*, in northeast Thailand: population changes of the snail intermediate host. Int J Parasitol.

[25] Namsanor J, Sithithaworn P, Kopolrat K, Kiatsopit N, Pitaksakulrat O, Tesana S (2015). Seasonal transmission of *Opisthorchis viverrini sensu lato* and a lecithodendriid trematode species in Bithynia siamensis goniomphalos snails in northeast Thailand. Am J Trop Med Hyg.

[26] Schell SC (1970). How to know the Trematode.

[27] Sithithaworn P, Yongvanit P, Tesana S, Pairojkul C, Darwin MK, Bernard F (2007). Liver flukes. Food-borne parasitic zoonoses.

[28] Kaewkes W, Kaewkes S, Tesana S, Laha T, Sripa B (2012). Fecal bacterial contamination in natural water reservoirs as an indicator of seasonal infection by *Opisthorchis viverrini* in snail intermediate hosts. Parasitol Int.

[29] Wang Y-C, Yuen R, Feng C-C, Sithithaworn P, Kim I-H (2017). Assessing the role of landscape connectivity on *Opisthorchis viverrini* transmission dynamics. Parasitol Int.

[30] Wang Y-C, Ho RCY, Feng C-C, Namsanor J, Sithithaworn P (2015). An ecological study of *Bithynia* snails, the first intermediate host of *Opisthorchis viverrini* in northeast Thailand. Acta Trop.

[31] Prasongwatana J, Laummaunwai P, Boonmars T, Pinlaor S (2013). Viable metacercariae of *Opisthorchis viverrini* in northeastern thai cyprinid fish dishes–as part of a rational program for control of *O. viverrini*-associated cholangiocarcinoma. Parasitol Res.

[32] Onsurathum S, Pinlaor P, Charoensuk L, Haonon O, Chaidee A, Intuyod K (2016). Contamination of *Opisthorchis viverrini* and *Haplorchis taichui* metacercariae in fermented fish products in northeastern Thailand markets. Food Control.

[33] Grundy-Warr C, Andrews RH, Sithithaworn P, Petney TN, Sripa B, Laithavewat L (2012). Raw attitudes, wetland cultures, life-cycles: Socio-cultural dynamics relating to *Opisthorchis viverrini* in the Mekong Basin. Parasitol Int.

[34] Kim CS, Smith JF, Suwannatrai A, Echaubard P, Wilcox B, Kaewkes S (2017). Role of socio-cultural and economic factors in cyprinid fish distribution networks and consumption in Lawa Lake region, Northeast Thailand: novel perspectives on *Opisthorchis viverrini* transmission dynamics. Acta Trop.

[35] R Core Team (2020). R: a language and environment for statistical computing.

[36] Ares G, Gámbaro A (2007). Influence of gender, age and motives underlying food choice on perceived healthiness and willingness to try functional foods. Appetite.

[37] Dube C, Ribble CS, Kelton D, McNab B (2011). Introduction to network analysis and its implications for animal disease modelling. Rev Sci Tech.

[38] Csardi G, Nepusz T (2006). The igraph software package for complex network research. InterJournal complex systems.

[39] Andrews RH, Sithithaworn P, Petney TN (2008). *Opisthorchis viverrini*: an underestimated parasite in world health. Trends Parasitol.

[40] Toledo R, Fried B (2005). Echinostomes as experimental models for interactions between adult parasites and vertebrate hosts. Trends Parasitol.

[41] Vinh HQ, Phimpraphai W, Tangkawattana S, Smith JF, Kaewkes S, Dung DT (2017). Risk factors for *Clonorchis sinensis* infection transmission in humans in northern Vietnam: a descriptive and social network analysis study. Parasitol Int.

[42] Phimpraphai W, Tangkawattana S, Kasemsuwan S, Sripa B (2018). Social influence in liver fluke transmission. Adv Parasitol.

[43] Ong X, Wang Y-C, Sithithaworn P, Grundy-Warr C, Pitaksakulrat O (2016). Dam influences on liver fluke transmission: fish infection and human fish consumption behavior. Ann Am Assoc Geogr.

[44] Pinlaor S, Onsurathum S, Boonmars T, Pinlaor P, Hongsrichan N, Chaidee A (2013). Distribution and abundance of *Opisthorchis viverrini* metacercariae in cyprinid fish in northeastern Thailand. Korean J Parasitol.

[45] Doreian P, Conti N (2012). Social context, spatial structure and social network structure. Soc Networks.

[46] Stacciarini J-M, Vacca R, Mao L (2018). Who and where: A socio-spatial integrated approach for community-based health research. Int J Environ Res Public Health.

[47] Sripa B, Tangkawattana S, Sangnikul T (2017). The Lawa model: a sustainable, integrated opisthorchiasis control program using the EcoHealth approach in the Lawa Lake region of Thailand. Parasitol Int.

[48] Sengupta ME, Hellström M, Kariuki HC, Olsen A, Thomsen PF, Mejer H (2019). Environmental DNA for improved detection and environmental surveillance of schistosomiasis. Proc Natl Acad Sci USA.

