# Supplementary material for: Examining ecosystem (dis-)services on liver fluke infection in rural Northeast Thailand
Source: Infect Dis Poverty. 2023 Apr 19;12:38. doi: 10.1186/s40249-023-01079-y (PMC10114451; doi:10.1186/s40249-023-01079-y)
Supplement: Supplementary file 1 — Additional file 1: Table S1. Snail species composition (%) in the surrounding water bodies of BN and BT villages. Table S2. Potential influences of perceptual and behavioral factors on consumption habits of BN and BT villagers. Consumption frequency was based on all three raw fish dishes consumed. Odds ratio for the analysis of willingness to avoid consumption and O. viverrini (O.v.) infection status was included for the relation that was statistically significant. Table S3. Potential influences of reasons of consumption (comparing five individual reasons vs. two reasons of ecosystem services), and perceptual and behavioral factors on consumption frequencies of BN and BT villagers. Consumption frequency was based on higher risk raw fish dishes, koi pla and pla som. Table S4. Potential influences of perceptual and behavioral factors, and network indices of degree centrality and betweenness centrality on consumption habits of BN and BT villagers. Consumption frequency wasbased on all three raw fish dishes consumed. Table S5. Potential influences of ecosystem services, perceptual and behavioral factors, and network indices of degree centrality and betweenness centrality on consumption habits of BN and BT villagers. Consumption frequency was based on all three raw fish dishes consumed. [file 40249_2023_1079_MOESM1_ESM.docx]

**Supplementary materials**

**Table S1.** Snail species composition (%) in the surrounding water bodies of BN and BT villages.

| Taxa | BN | BT |
| --- | --- | --- |
| *Bithynia* spp. | 95.9 | 98.3 |
| *Clea* spp. | 0.4 | 0.0 |
| *Filopaludina* spp. | 0.1 | 0.0 |
| *Melanoides* spp. | 1.3 | 0.0 |
| *Pomacea* spp. | 1.3 | 1.4 |
| *Trochotaia* spp. | 0.6 | 0.3 |

**Analysis of the potential influences various perceptual and behavioral factors had on consumption habits, using five individual consumption reasons (Table S2), in comparison with two consumption reasons of ecosystem services (i.e., cultural and provisioning) shown in Table 6 of the main text.**

The results in Table S2 showed that consumption frequency was motivated by it being the main source of protein (P = 0.039), and was positively associated with participation in food sharing activities (P = 0.006). For all the respondents from both villages, those who indicated ‘yes’ for eating raw fish as their main source of protein were likely to consume raw fish dishes 122.2 days/year more than other respondents who indicated ‘no’. Likewise, those who participated in food sharing activities were likely to consume 80.8 days/year more than other respondents who did not participate in food sharing. As for the associations between willingness to avoid consumption and various factors analyzed, statistical significances were detected for participation in food sharing activities. The odds of a respondent expressing willingness to avoid raw fish is 18% higher among those who shared raw fish dishes compared to those who did not participate in raw fish dish sharing (P = 0.001) when the reasons for consumption were analyzed as separate variables.

**Table S2.** Potential influences of perceptual and behavioral factors on consumption habits of BN and BT villagers. Consumption frequency was based on all three raw fish dishes consumed. Odds ratio for the analysis of willingness to avoid consumption and *O. viverrini* (*O.v.*) infection status was included for the relation that was statistically significant.

| Variable | Consumption frequency | | Willingness to avoid consumption | | *O.v.* infection status | |
| --- | --- | --- | --- | --- | --- | --- |
|  | Estimate | P-value | Estimate | P-value | Estimate | P-value |
| Importance as Isan culture | 6.68 ± 24.45 | 0.785 | -0.54 ± 0.41 | 0.184 | -0.54 ± 0.64 | 0.398 |
| Preference for taste | -5.46 ± 27.01 | 0.840 | -0.86 ± 0.46 | 0.063 | 0.08 ± 0.72 | 0.914 |
| Convenience | 23.84 ± 31.33 | 0.449 | -0.02 ± 0.49 | 0.967 | -0.03 ± 0.78 | 0.971 |
| Source of protein | 122.6 ± 58.47 | 0.039* | 13.61 ± 753.08 | 0.986 | 1.43 ± 1.18 | 0.224 |
| Social gathering | -32.81 ± 27.46 | 0.235 | 0.05 ± 0.43 | 0.901 | 1.07 ± 0.62 | 0.085 |
| Willingness to avoid consumption | -17.77 ± 28.1 | 0.529 | - | - | 0.89 ± 0.89 | 0.319 |
| Awareness of health consequences | -14.49 ± 84.88 | 0.865 | 1.4 ± 1.28 | 0.276 | 8.63 ± 497.59 | 0.986 |
| Participation in food sharing activities | 80.78 ± 28.77 | 0.006* | -1.69 ± 0.5 | 0.001* | 0.99 ± 0.76 | 0.195 |
| Degree centrality of food sharing | -27.23 ± 17.36 | 0.120 | 0.39 ± 0.27 | 0.148 | 0.16 ± 0.33 | 0.638 |
| Frequency of consumption | - | - | 0 ± 0 | 0.345 | 0 ± 0 | 0.195 |
| Gender – Male | 40.12 ± 42.02 | 0.342 | -0.83 ± 0.67 | 0.217 | -0.33 ± 1.09 | 0.765 |

**Analysis of the potential influences various perceptual and behavioral factors had on consumption frequency of higher risk raw fish dishes, using five individual consumption reasons vs. two consumption reasons of ecosystem services (i.e., cultural and provisioning).**

As *koi pla* and *pla som* are higher risk raw fish dishes, the potential influence of the five individual reasons for consumption, perceptual, behavioral and demographic factors on the combined consumption frequency of these two dishes was investigated. Two sets of linear mixed effect models were performed following equation (3) in the main text, where the frequency of consumption of *koi pla and* *pla som* combined was used as dependent variable *Y*, and two sets of fixed effect variables *X* were used, one keeping the reasons for consumption as individual variables, the other categorizing them into their respective ecosystem services*.* A random effect *Z* was included to represent and account for potential variations between the two villages.

The results in Table S3 revealed statistical significance in the relationship between gender and consumption frequency for both sets of analyses. Male villagers were likely to consume *koi pla* and *pla som* 61.4 days/year more than female villagers for consumption reasons assessed as individual variables, and 57.5 days/years more than female villagers for consumption reasons assessed as ecosystem services. Participation in food sharing activities was also found to have statistically significant correlation with consumption frequency. Villagers who participated in food sharing activities were likely to consume the combination of *koi pla* and *pla som* 27.8 days/year more than villagers who did not.

Consumption for the reason of main source of protein showed statistically significant correlation to an increased consumption frequency. However, upon investigating the respondents who consumed raw fish for the reason of main source of protein, the data revealed that the above relationship was likely driven by one individual who consumed these two higher risk dishes daily, while the rest of the respondents either do not consume those dishes or consume only once a year. Therefore, statistical inferences would not be drawn from this set of analysis for the main text, and reliable insights should only be drawn with further substantiation of additional observations to minimize the influence of skewed observations.

**Table S3.** Potential influences of reasons of consumption (comparing five individual reasons vs. two reasons of ecosystem services), and perceptual and behavioral factors on consumption frequencies of BN and BT villagers. Consumption frequency was based on higher risk raw fish dishes, *koi pla* and *pla som*.

| Consumption reason as individual variables | | | Consumption reasons by ecosystem services | | |
| --- | --- | --- | --- | --- | --- |
| Variable | Estimates | P-value | Variable | Estimates | P-value |
| Preference for taste | -3.16 ± 11.65 | 0.786 | Cultural ecosystem services | 0.46 ± 21.36 | 0.983 |
| Importance as Isan culture | -5.62 ± 10.65 | 0.599 | Provisioning ecosystem services | 18.28 ± 12.51 | 0.147 |
| Social gathering | -14.43 ± 11.51 | 0.213 | Willingness to avoid consumption | 23.48 ± 12.49 | 0.063 |
| Convenience | 6.57 ± 13.76 | 0.634 | Awareness of health consequences | -12.84 ± 38.15 | 0.737 |
| Source of protein | 94.13 ± 25.68* | 0.000* | Participation in food sharing activities | 27.81 ± 12.58* | 0.029* |
| Willingness to avoid consumption | 13.98 ± 12.28 | 0.258 | Degree centrality of food sharing | -11.56 ± 7.83 | 0.143 |
| Awareness of health consequences | -8.64 ± 36.98 | 0.816 | Frequency of consumption | - | - |
| Participation in food sharing activities | 22.9 ± 12.02 | 0.060 | Gender – Male | 57.5 ± 18.41* | 0.002* |
| Degree centrality of food sharing | -4.75 ± 7.87 | 0.547 |  |  |  |
| Gender – Male | 61.42 ± 18.15* | 0.001* |  |  |  |

**Additional analyses in extension to Table 6 in the main text and Table S2 above.**

Tables S4 and S5 show the results of additional analyses performed in extension to Table S2 above and Table 6 in the main text respectively where an additional independent variable of betweenness centrality was included. It should be noted that the inclusion of betweenness centrality as an independent variable markedly reduced the number of complete observations available for the regression analyses to 46, compared to 107 complete observations available when betweenness centrality was not included as an independent variable. This was due to the nature of the betweenness centrality measure. If nodes were not linked to any other nodes, they would have the record of ‘NA’ for betweenness centrality. These ‘NA’ data points were then excluded from the regression model due to their incompleteness. We therefore posit to only include degree centrality in the main text as a comparatively more relevant indicator of connectedness, without compromising the robustness of the regression model and the reliability of the insights that could be drawn from the results.

The results in Tables S4 and S5 therefore must be interpreted cautiously with the above limitations borne in mind. Notably, none of the relationships between each of the three dependent variables and degree and betweenness centrality measures was statistically significant. Nevertheless, the results show that consumption for preference for taste was significantly correlated with willingness to avoid raw fish consumption (Table S4). The odds of a villager expressing willingness to avoid raw fish consumption for the preference for taste was 0.24 of the odds of a villager expressing willingness to avoid raw fish consumption but do not consume for the reason of preference for taste. This means that among the villagers who express willingness to avoid raw fish consumption, the odds of a villager not consuming for preference for taste is 4.2 times that of a villager consuming for preference for taste. Consumption motivated by the derivation of cultural ecosystem services was also statistically significantly correlated with consumption frequency (Table S5). Villagers whose consumptions were motivated by the derivation of cultural ecosystem services were likely to consume 259.3 days/year less than those who were not. Notwithstanding the statistically significant correlations detected, statistical evidence should not be inferred from these results due to the limited number of available observations.

**Table S4.** Potential influences of perceptual and behavioral factors, and network indices of degree centrality and betweenness centrality on consumption habits of BN and BT villagers. Consumption frequency was based on all three raw fish dishes consumed.

| Variable | Consumption frequency | | Willingness to avoid consumption | | *O.v.* infection status | |
| --- | --- | --- | --- | --- | --- | --- |
|  | Estimate | P-value | Estimate | P-value | Estimate | P-value |
| Preference for taste | -40.73 ± 43.21 | 0.35 | -1.44 ± 0.73  (Odds ratio = 0.24) | 0.05* | -0.48 ± 1.21 | 0.69 |
| Importance as Isan culture | 12.59 ± 41.08 | 0.76 | -1.04 ± 0.88 | 0.24 | -1.75 ± 1.17 | 0.14 |
| Social gathering | 37.34 ± 37.18 | 0.32 | -0.47 ± 0.68 | 0.48 | 1.44 ± 1.03 | 0.16 |
| Convenience | -19.95 ± 44.47 | 0.66 | 0.8 ± 0.85 | 0.35 | 0.09 ± 0.98 | 0.93 |
| Source of protein | -69.19 ± 117.91 | 0.56 | 14.26 ± 689.67 | 0.98 | 15.85 ± 1477.07 | 0.99 |
| Willingness to avoid consumption | -0.23 ± 39.15 | 1.00 | - | - | 0.41 ± 1.1 | 0.71 |
| Awareness of health consequences | -16.19 ± 120.54 | 0.89 | 15.15 ± 567.8 | 0.98 | 10.8 ± 1475.4 | 0.99 |
| Participation in food sharing activities | 100.13 ± 54.1 | 0.07 | -13.12 ± 575.37 | 0.98 | 13.83 ± 1492.43 | 0.99 |
| Degree centrality of food sharing | -7.98 ± 34.01 | 0.82 | -0.2 ± 0.71 | 0.78 | 0.66 ± 0.75 | 0.38 |
| Betweenness centrality of food sharing | -2.91 ± 5.48 | 0.60 | 0.22 ± 0.29 | 0.44 | -0.1 ± 0.12 | 0.38 |
| Frequency of consumption | - | - | 0 ± 0 | 0.30 | 0 ± 0 | 0.37 |
| Gender – Male | -44.8 ± 60.57 | 0.46 | -0.59 ± 1.04 | 0.57 | -0.53 ± 1.62 | 0.75 |

**Table S5.** Potential influences of ecosystem services, perceptual and behavioral factors, and network indices of degree centrality and betweenness centrality on consumption habits of BN and BT villagers. Consumption frequency was based on all three raw fish dishes consumed.

| Variable | Consumption frequency | | Willingness to avoid consumption | | *O.v.* infection status | |
| --- | --- | --- | --- | --- | --- | --- |
|  | Estimate | P-value | Estimate | P-value | Estimate | P-value |
| Cultural ecosystem services | -259.26 ± 104.85 | 0.02* | -13.67 ± 12537.24 | 1.00 | 13.1 ± 12537.3 | 1.00 |
| Provisioning ecosystem services | -50.87 ± 35.53 | 0.16 | 0.2 ± 0.62 | 0.75 | 1.1 ± 0.8 | 0.17 |
| Willingness to avoid consumption | -5.98 ± 33.33 | 0.86 | - | - | 1.13 ± 0.92 | 0.22 |
| Awareness of health consequences | -37.97 ± 103.55 | 0.72 | 13.91 ± 12537.29 | 1.00 | 10.49 ± 12537.3 | 1.00 |
| Participation in food sharing activities | 80.61 ± 47.11 | 0.10 | -13.76 ± 5011.39 | 1.00 | 12.98 ± 4719.62 | 1.00 |
| Degree centrality of food sharing | -11.91 ± 30.45 | 0.70 | -0.15 ± 0.64 | 0.81 | 0.56 ± 0.55 | 0.31 |
| Betweenness centrality of food sharing | -2.11 ± 5.04 | 0.68 | 0.23 ± 0.24 | 0.33 | -0.09 ± 0.11 | 0.37 |
| Frequency of consumption | - | - | 0 ± 0 | 0.81 | 0 ± 0 | 0.37 |
| Gender – Male | -21.24 ± 49.11 | 0.67 | 0.07 ± 0.79 | 0.93 | 1.01 ± 1.17 | 0.39 |
